# Interactions between metabotropic glutamate and CB1 receptors: implications for mood, cognition, and synaptic signaling based on data from mGluR and CB1R-targeting drugs

**DOI:** 10.1007/s43440-024-00612-6

**Published:** 2024-06-28

**Authors:** Katarzyna Stachowicz

**Affiliations:** Department of Neurobiology, Maj Institute of Pharmacology, Polish Academy of Sciences, Smętna 12, Kraków, 31-343 Poland

**Keywords:** CB1Rs, mGluRs, Cannabinoid CB1, Metabotropic glutamate receptors, Mood, Cognition

## Abstract

Metabotropic glutamate receptors (mGluRs) are part of the G protein-coupled receptors (GPCRs) family. They are coupled to G_αq_ (group I) or G_i/o_ (groups II and III) proteins, which result in the generation of diacylglycerol (DAG) and inositol 1,4,5-triphosphate (IP_3_) or the inhibition of adenylyl cyclase, respectively. mGluRs have been implicated in anxiety, depression, learning, and synaptic plasticity. Similarly, CB1 cannabinoid receptors (CB1Rs), also GPCRs, play roles in cognitive function and mood regulation through G_αi/o_–mediated inhibition of adenylyl cyclase. Both mGluRs and CB1Rs exhibit surface labeling and undergo endocytosis. Given the similar cellular distribution and mechanisms of action, this review complies with fundamental data on the potential interactions and mutual regulation of mGluRs and CB1Rs in the context of depression, anxiety, and cognition, providing pioneering insights into their interplay.

## Introduction

Mood and cognitive disorders affect many patients with diagnosed mental illness, including those suffering from depression or anxiety [1]. In the clinic, the drugs of first choice in depression are monoamine oxidase inhibitors, selective serotonin reuptake inhibitors, or tricyclic antidepressants, among others [1]. However, the side effects associated with first-line drugs are severe, and treatment must be continued for more than a few weeks to see positive results. If these results are not achieved, treatment is extended, forcing a change in therapy [1]. As a solution to the problem, hope was seen in a mechanism discovered in the 1990s involving glutamate and neurotransmission based on receptors for glutamate, including metabotropic glutamatergic receptors [1]. According to glutamatergic theory, mental disorders, including depression, can be a consequence of dysregulation of glutamate levels in the brain, its excessive release into the synaptic space resulting in neurotoxicity, which leads to dendritic endings atrophy, and behavioral changes [1]. However, the mGluRs ligands which are promising in basic research have not reached the clinic yet. A parallel discovery of recent years is the demonstration that mGlu receptors form homo- and heterodimers with each other but also with other GPCRs [2–4] and oligomers [4]. Heterodimers include mGluR1/5, mGluR2/3, mGluR2/4, and mGluR2/7 [5, 6]. During hetero-dimerization, the receptor adopts various conformational states, including inactive, intermediate inactive, intermediate active, and active [4]. These changes are attributed to conformational alterations in the Venus flytrap domains (VFDs) and significant rearrangements in the transmembrane domains [4]. The transmembrane domains transition from an inactive symmetric dimer to an active asymmetric dimer [4]. More details can be found in Wang et al. [4]. Furthermore, functional interaction between mGlu2/3-5-HT2A, mGlu4-5-HT1A, and also mGlu2-TrkB receptors has been demonstrated [2–4, 7, 8]. The discovery of such behavior of mGluRs receptors raises hopes for finding a partner that will accelerate and enhance the action of mGluRs ligands while depriving them of undesired actions. From our long-term studies, we have concluded that such a partner in mental health problems may be a components of arachidonic acid pathways and CB1Rs [1]. Surprisingly, there are only a few studies on the interactions of mGluRs and CB1Rs receptors. Therefore, the main purpose of this review is to highlight the potential points of interaction between mGluRs and CB1R receptors at both the functional and cellular signaling levels. This represents a novel approach to the subject and may contribute to identifying new targets for central nervous system pharmacotherapy.

## Structure and signaling of metabotropic glutamate receptors

**Fig. 1:**
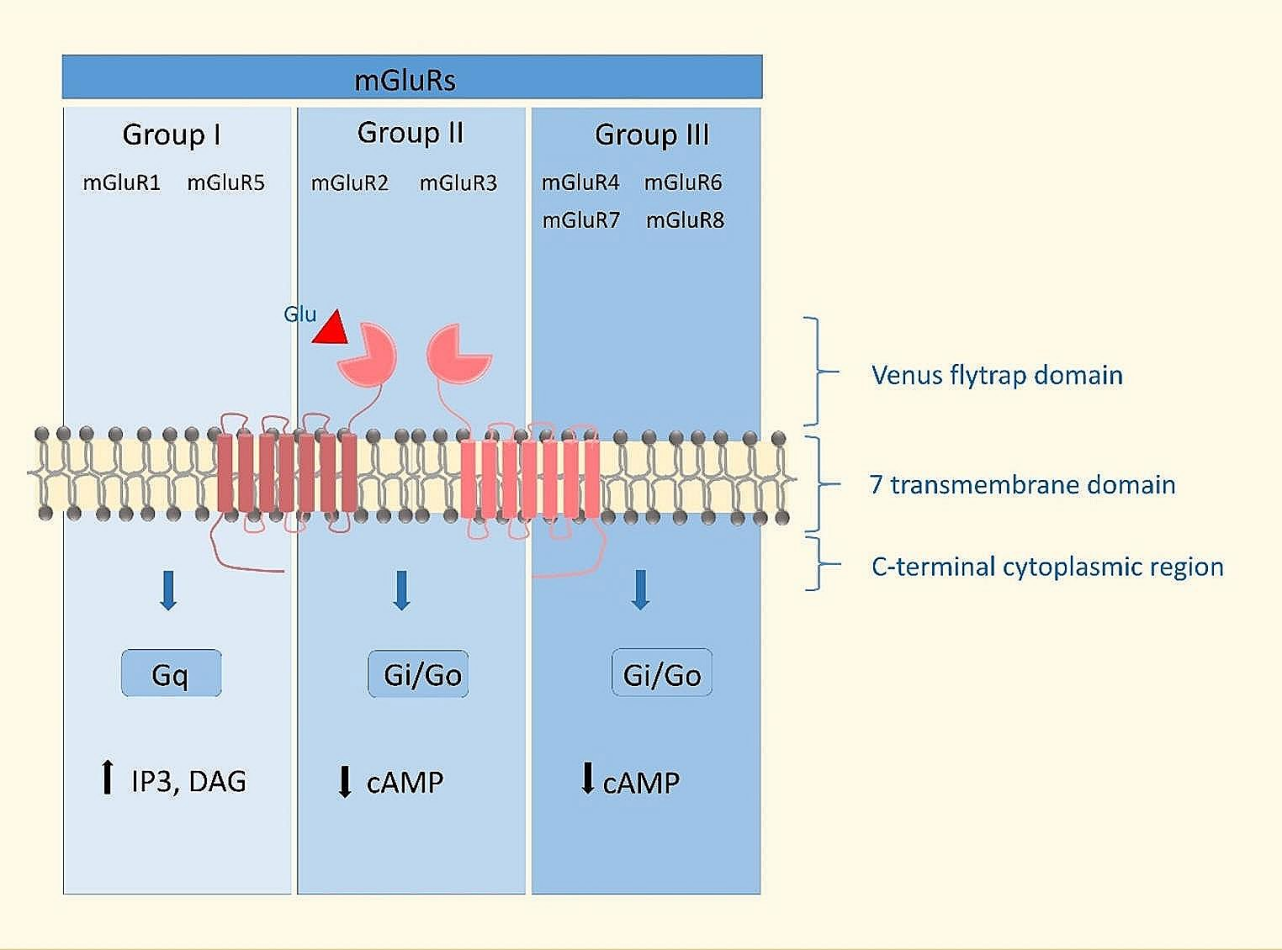
Schematic of mGlu receptor structure, classification, and mechanisms involved in their function

Based on sequence similarity, pharmacology, and coupling to second messenger pathways, metabotropic glutamate receptors (mGluRs) are divided into mGluRI, mGluRII, and mGluRIII [9]. The mGluR receptors are activated by glutamate (Glu), a non-essential amino acid and a major excitatory neurotransmitter [9]. The mGluRs family of receptors are localized within the synapse pre-synaptically, post-synaptically, or extra-synaptically to control the release of neurotransmitters locally or to regulate from different sources the neurotransmitters released [5]. The classification, structure, and signaling of mGluR are shown in Fig. 1.

The mGlu receptors are seven-transmembrane proteins. The mGluRs feature a substantial extracellular N-terminal domain known as the VFD, which contains the glutamate binding site formed by two lobes [10]. Upon ligand binding, conformational changes are transmitted through the cysteine-rich domains to the C-terminal tail [10, 11]. The two VFDs are dimerized for the agonist to bind [10]. Three conformational states of VFDs have been shown to exist: open-open (inactive), open-closed, and closed-closed [10, 11]. The C-terminus of the mGluR dictates the G protein coupling, which varies depending on the specific group of activated mGluRs:

Group I mGluR consists of two members, mGluR1 (splice variants: a, b, c, d, e, f) and mGluR5 (a, b), extensively expressed in the brain [9, 10, 12]. The mGluR1 expression was found in the cortex, olfactory bulb, globus pallidus, and thalamus [10, 12]. The mGluR5 is expressed in the cortex, hippocampus, olfactory bulb, striatum, nucleus accumbens, and superficial spinal cord dorsal horn [10, 12]. The mGluRI are primarily localized postsynaptically, and their activation is coupled with G_αq_ proteins, stimulation of phospholipase C (PLC), and the formation of diacylglycerol (DAG) and inositol-1,4,5-triphosphate (IP_3_) [9, 10, 12]. The activation of those downstream signaling is coupled with the release of Ca^2+^ from intracellular stores, activation of protein kinase C (PKC) and phospholipase D (PLD), phospholipase A2 (PLA2), and mitogen-activated kinases (MAPKs) [9, 10, 12]. Activation of mGluR1/5 is linked to the signal associated with a range of downstream effectors e.g.: the Homer proteins, N-methyl-D-aspartate receptors (NMDARs), Jun kinase, extracellular receptor kinase (ERK), MAPK/ERK, mammalian target of rapamycin (MTOR)/p70 S6 kinase [9, 10, 12].

Group II mGluRs include mGluR2 and mGluR3 (GRIM3A2, GRIM3A4, GRIM3A2A3), which are mainly localized presynaptically, acting as autoreceptors to inhibit the release of Glu or γ-aminobutyric acid (GABA) [10, 12]. mGluR2 is expressed primarily in the cerebral cortex and olfactory bulb, while mGluR3 is expressed throughout the brain [12]. Activation of mGluRII couples to G_i/o_ proteins induces adenylyl cyclase inhibition and decreases cAMP levels [12]. The same mechanism is mediated by mGluRIII. Group III mGluRs include mGluR4, mGluR6 (a, b, c), mGluR7 (a, b, c, d, e), mGluR8 (a, b, c) and, like mGluRII, are mainly localized presynaptically, acting as autoreceptors to inhibit Glu or GABA release [10, 12]. Expression of mGluRIII has been documented in the whole brain, mGluR6 in the retina, and mGluR4 was additionally detected in the cerebellum [12]. More precisely, mGluR4 is localized in the olfactory bulb, cortex, hippocampus, hypothalamus, and cerebellum, among others [13]. mGluR7 is expressed in sensory afferent pathways, olfactory bulb, hippocampus, and hypothalamus [13]. mGluR8 was found in the retina, olfactory bulb, in the cortex (piriform cortex, anterior cingulate cortex), and the amygdala [13]. In addition to G_i/o_ -protein-related signaling after activation of group III receptors, as described earlier, the G_βγ_-protein-related pathway can also be activated, which is connected with stimulation of G-protein inward rectifying K^+^ channels (GIRK) [13]. Activation of mGluR III is linked to the signal associated with such downstream effectors as γ-aminobutyric acid (GABA), Pi3K/AKT, MAPK/ERK, brain-derived neurotrophic factor (BDNF), tumor necrosis factor-alpha (TNF-α), inducible nitric oxide synthase (iNOS) [13]. Furthermore, as it states mGluR7 it has the potential to modulate NMDARs, to prevent the NMDA-mediated excitotoxicity of cholinergic neurons [13].

As GPCRs, mGluR receptors undergo desensitization, internalization, and endocytosis [9]. Prolonged or repeated exposure of receptors to agonist stimulation has been documented to lead to receptor desensitization, described as the loss of receptor response [9, 14]. While the exact mechanism of desensitization remains unclear, it is believed to be an adaptive process that prevents overstimulation and helps filter information [9]. In the case of mGluR1, phosphorylation and G-protein-coupled receptor kinases (GRKs) are thought to play a crucial role [9, 14]. Phosphorylation, as an example of post-translational modification, has been documented in mGluR1a and mGluR1b via PKC, leading to receptor desensitization [9]. A similar mechanism involving serine/threonine residues has been found in the case of mGluR5 [9]. Conversely, PKA has the potential to inhibit the desensitization mechanism [9]. When GRKs act through phosphorylation, arrestins are activated to uncouple the G-protein from the receptor, resulting in desensitization [9, 14]. The formed GPCR/arrestin complex, which has become phosphorylated, is directed to clathrin-coated pits, and the GPCR is dephosphorylated and recycled to the plasma membrane or down-regulated [14]. The internalized receptor is recycled back to the cell surface in a process known as resensitization [15, 16].

### Involvement of metabotropic glutamate receptors in mood and cognition

Since stress-related disorders, depression, and anxiety are associated with the overactivation of the glutamatergic system and Glu-induced excitotoxicity, much research has been directed toward compounds that act through receptors for glutamate, among which there are several mGluRs ligands. Thanks to these compounds, it is possible to trace the activated mechanisms during the mentioned diseases [12, 17–21]. In addition, there is evidence that Glu dysregulation is associated with cognitive impairment and memory decline [21]. Postmortem studies of depressed patients have shown increased levels of Glu in the brain, deregulated levels in cerebrospinal fluid and plasma, and volume changes in areas of the brain supplied with Glu neurons (amygdala, hippocampus, cortex), among others [19, 21]. Antidepressant-like or anxiolytic-like effects have been documented in animal studies across a range of mGluR ligands. Among them, antidepressant-like action showed mGluRI antagonists, negative allosteric modulators (NAMs), inverse agonists, mGluRII agonists, antagonists, NAMs, mGluRIII agonists, positive allosteric modulators (PAMs), and allosteric agonists [17, 21]. Regardless of their activity, through various mechanisms, these ligands have the task pre- or post-synaptically of restoring homeostasis of the Glu system in the brain and synapses, which is reflected in the behaviors. Details of the mGluRs ligands’ activity in animal models are given in Table 1.

**Table 1:** Behavioral effects of selected mGluRs ligands in depressive-like, anxiety-like, and cognitive studies

Behavioral effects	ligand	Dose	Model/test	Ref.
Depressive symptoms studies				
Antidepressant-like	Group I: GRN529 (mGluR5 NAM)	0.1–30 mg/kg po/acute	TST/FST (mice, rats)	[22]
Antidepressant-like	MTEP (mGluR5 antagonist/ NAM)	0.3-3 mg/kg mice 1–10 mg/kg rats *ip*/acute and 14 days	TST/FST (mice, rats)	[23]
Antidepressant-like	DSR98776 (mGluR5 inverse agonist)	1–3 mg/kg rats/7 days 10–30 mg/kg/mice/ acute	FST (mice, rats)	[24]
Antidepressant-like	**Group II**: MGS0039 (mGluR2/3 antagonist)	0.3-3 mg/kg *ip*/acute	TST/FST (mice, rats)	[25]
Antidepressant-like	Ro4491533 (mGluR2/3 NAM)	1-100 mg/kg *po*/acute	TST/FST (mice, rats)	[26]
Antidepressant-like	VU6010572 (mGluR3 NAM)	3 mg/kg *ip*/ acute	TST (mice)	[27]
Pro-depressive-like	**Group III**: LSP42022 (mGluR4 agonist)	0.5, 1 mg/kg *ip*/acute	TST/FST (mice, rats)	[28]
Antidepressant-like	AMN082 (mGluR7 allosteric agonist)	1, 3, 6 mg/kg *ip*/acute	FST (mice, confirmed in mGluR7 KO)	[29]
Antidepressant-like	RSPPG (mGluR8 agonist)	7.5, 15 nmol/rat/acute intrahippocampal	FST (rats)	[30]
**Anxiety symptoms studies**				
Anxiolytic-like	**Group I**: AIDA (mGluRI antagonist)	0.5-2 mg/kg *ip*/acute	Vogel conflict (rats)	[31, 32]
Anxiolytic-like	**Group II**: LY341495 (mGluR2/3 antagonist)	0.1, 0.3, 1 mg/kg *ip*/ acute	SIH (mice)	[33]
Anxiolytic-like	**Group III**: ACPT-I (mGluRIII agonist)	7.5 nmol/µl/rat/acute basolateral amygdala	SIH (mice)	[34]
Anxiolytic-like	PHCCC (mGluR4 PAM)	6.25, 12.5 nmol/µl rat/acute basolateral amygdala	Vogel conflict (rats)	[35–37]
Anxiolytic-like	AMN082 (mGluR7 allosteric agonist)	3, 6 mg/kg *ip*/acute	SIH (mice)	[37]
**Cognitive effects**				
Improved cognitive performance and reduced Aβ pathology	**Group I**: CTEP (mGluR5 NAM)	2 mg/kg *ip*/acute vs. chronic (12 weeks)	APPswe/PS1DE9 and 3X-Tg mice	[38]
Reversed learning and memory impairment, decreased level of Aβ	**Group II**: BCI632 (mGluRII antagonist) active metabolite of BCI-838	prodrug/*po*/chronic (3 months)	AD model (mice)	[39]
Impairment of acquisition, consolidation and retrieval of long-term memory	**Group III**: AMN082 (mGluR7 allosteric agonist)	1.25, 2.5, 5 mg/kg *ip*/ acute	PA (mice, rats)	[40]
Facilitation of extinction and inhibition of reinstatement after morphine	AMN082 (mGluR7 allosteric agonist)	1 and 5 µg/0.5 µl bilaterally nucleus accumbens	CPP (rats)	[41]

AD– Alzheimer’s disease, CPP - conditioned place preference (morphine-induced), FST– forced swim test, PA - passive avoidance, SIH– stress-induced hyperthermia, TST–tail suspension test

The forced swim (FST) test and tail suspension test (TST) are validated screening models to search for the antidepressant-like activity of chemical compounds in rodents. Antidepressant-like effects in TST were confirmed in GRN529 (mGluR5 NAM), MTEP (mGluR5 antagonist and NAM), MGS0039 (mGluR2/3 antagonist), Ro4491533 (mGluR2/3 NAM), VU6010572 (mGluR3 NAM) [22, 23, 25, 26, 28]. Furthermore, in FST, antidepressant-like potential was found as it states to GRN529, MTEP, DSR98776 (mGluR5 inverse agonist), MGS0039, Ro4491533, LSP42022 (mGluR4 agonist), AMN082 (mGluR7 allosteric agonist), RSPPG (mGluR8 agonist), and PHCCC (mGluR4 PAM) [22–30, 34, 35, 36]. Pro-depressive-like effects were found after LSP42022 (mGluR4 agonist) application [28]. Besides its activity in depression, mGluRs ligands were effective as anxiolytics in rodents. One of the first reports in the field was anxiolytic-like activity found after AIDA, LY456236 application (mGluR1 antagonists), and PHCCC [31, 32]. AIDA increased the number of shocks accepted in the conflict drinking Vogel test in a specific manner, while it did not affect rats’ threshold current and water intake [31]. Further studies based on stress-induced hyperthermia tests documented the anxiolytic-like potential of LY341495 (mGluR2/3 antagonist), ACPT-I (mGluRIII agonist), and AMN082 (mGluR7 allosteric agonist) [33–37].

As documented in animal studies, the mGlu receptors are also involved in cognitive processes [42, 43]. mGluRs ligands are known for positive and negative interactions with memory parameters in rodents [38–41]. Mental changes are best studied in models of Alzheimer’s disease (AD). Hence, the significant involvement of mGluRs in memory-related mechanisms has been documented [12, 19]. In vivo, the neuroprotective effects of mGluRI, mGluRII, and mGluRIII ligands have been reported [19]. Details can be found in Table 1. The potential of mGluRs in cognition is not only based on neuroprotection but they have been documented to be involved in reducing Aβ plaques and Aβ oligomers, as well as improving cognitive performance [12, 44]. Kim et al. [39], using APP transgenic mice forming Aβ-oligomers (APP E693Q), documented that the group II mGluR antagonist (BCL-632) has strong potential to reverse transgene-associated amnesia, reduced anxiety, and Aβ mono- and oligomers. Mice had significantly better memory as measured in contextual conditioning and novel object recognition tasks [39]. Mechanisms of hippocampal neurogenesis have been speculated to be beneficial in this case [39].

Given the vast amount of preclinical research, mGlu receptors are undoubtedly involved in the mechanism of anxiety, depression, or memory-related changes [17, 45, 46]. However, these findings do not translate into the clinic, as described by Jiang et al. [17] and White et al. [18]. Therefore, looking for common pathways and actions with other neurotransmission systems that may interfere with the observed effects is essential.

### Involvement of cannabinoid CB1 receptors in mood and cognition

Endogenous cannabinoids (eCBs), the enzymes involved in their synthesis and metabolism, and G-protein-coupled cannabinoid receptors (GPCRs), namely CB1 and CB2, are part of the endogenous cannabinoid system [47]. CB1 receptors (CB1R) are distributed in the brain mainly at presynaptic terminals. Their activation induces inhibition of the release of neurotransmitters, which are gamma-aminobutyric acid (GABA), glutamate (Glu), serotonin, dopamine, norepinephrine, and acetylcholine [47]. Postsynaptically localized CB1Rs regulate the activity of selected ion channels and N-methyl-D-aspartate receptors (NMDARs) [47]. Through these actions, CB1Rs have been described as active regulators of mood and cognition [48, 49]. CB1Rs are known for their actions in pain and nociception, but in depression and cognition, the results of their actions are distinct, and the conclusion is complicated. Cannabinoids present an U-shaped dose-response curve which is widely described in the literature [50, 51]. Cannabinoids have been shown to have anxiolytic-like effects at low doses and anxiogenic-like effects at high doses [50, 51]. The effects have been observed both in preclinical and clinical studies [50, 51]. In addition, the use of cannabinoids for depression has been shown to carry a risk of side effects such as psychosis, cannabis-use disorders, increased depression, or impairment of attention during the abstinence period [48, 52]. Increased CB1R expression in the prefrontal cortex of depressed/suicidal patients and elevated serum levels of anandamide (AEA) or 2-arachidonoylglycerol (2-AG) were found in patients with minor depression and reduced levels in patients with major depression [48]. It has been shown that a single nucleotide polymorphism in CB1R may be associated with susceptibility to depression [48]. There are quite a few preclinical and clinical studies on the topic, and they are presented in more detail in Table 2.

**Table 2:** Behavioral effects of selected CB1Rs ligands in depressive-like, anxiety-like, and cognitive studies

Behavioral effects	ligand	Dose	Model/test	Ref.
Depressive symptoms studies				
Pro-depressant	Rimonabant (CB1Rs antagonist)	5 mg/day, 20 mg/day/ two years	Clinic/obesity	[53]
Antidepressant-like	HU210 (CB1Rs agonist)	50 µg/kg	FST, SPT (mice)	[54]
Antidepressant-like	WIN55212-2 (CB1Rs agonist)	0.5 mg/kg *ip*/four weeks	UMS (rats)	[55]
Antidepressant-like	CP55940 (CB1Rs agonist)	0.03–0.3 mg/kg *ip*/acute	FST (rats)	[56]
Antidepressant-like	AM404 (eCB uptake inhibitor)	0.3-3 mg/kg *ip*/acute	FST (rats)	[56]
Antidepressant-like	URB597 (FAAH inhibitor)	0.1–0.3 mg/kg *ip*/acute	FST (rats)	[56]
Antidepressant-like	CBD	0.5–50 mg/kg *ip*/acute	FST, OBX (rats, mice)	[57]
**Anxiety symptoms studies**				
Anxiogenic-like	AM251 (CB1Rs antagonist)	1.5-3 mg/kg *ip*/acute	EPM (mice)	[58]
Anxiolytic-like	AM251 (CB1Rs antagonist)	1-100 pmol/rat/midbrain dorsolateral periaqueductal gray	PM (rats/PAG)	[59]
Anxiolytic-like	Rimonabant (CB1Rs antagonist)	0.1-3 mg/kg *ip*/acute	Vogel conflict (rats)	[60]
Anxiolytic-/ Anxiogenic-like	CBD	0.5–50 mg/kg *ip*/acute	EPM (rats, mice)	[57]
**Cognitive effects**				
Improved recognition memory	AM251 (CB1Rs antagonist)	1, 2.5, 5 mg/kg *ip*/acute	ORT (rats)	[61]
Disruption of reconsolidation	AM251 (CB1Rs antagonist)	300 ng per 0.5 ml per side/ basolateral amygdala/acute	AFC BLA-infusion (rats)	[62]
Increased conditioning freezing	URB597 (FAAH inhibitor)	30 ng per 0.5 ml per side/ basolateral amygdala/acute	AFC BLA-infusion (rats)	[62]

AFC - auditory fear conditioning, EPM - elevated plus maze, FST - forced swim test, OBX - olfactory bulbectomy, ORT - object recognition test, SPT - sucrose preference test, UMS - unpredictable mild stress

Pro-depressive effects of rimonabant (CB1R antagonist) were found in incidents in clinics for obesity treatment [53]. However, most of the preclinical research documented the antidepressant-like potential of CB1R ligands. Among them, antidepressant-like potential showed HU210, WIN55212-2, CP55940 (CB1Rs agonists), AM404 (eCB uptake inhibitor), and URB597 (FAAH inhibitor) [52–55]. Cannabidiol (CBD) is an extract of the Cannabis plant, without psychotomimetic activity and low affinity for CB1 and CB2 receptors [57]. CBD was shown to be potent as an antidepressant in animal models in doses between 7 and 200 mg/kg [57]. Depending on studies tested, its antidepressant-like potential was connected with 5-HT1A receptors or BDNF [57]. As it states to anxiety, CB1Rs antagonist– AM251 - showed anxiogenic-like effects in mice, while in rats, anxiolytic-like [58, 59]. Similar anxiolytic-like effects showed rimonabant in the Vogel conflict test in rats [60]. CBD was shown to be potent as anxiogenic- or anxiolytic-like [57]. Discrepancies are due to doses used in a case of CD1Rs ligands, presenting contrast effects.

AM251 injected into rats improved recognition memory (acquisition and consolidation) in object recognition tests at a dose of 1 mg/kg, while higher doses (2.5 and 5 mg/kg) did not have such properties [61]. The same compound was shown to impair the reconsolidation of Pavlovian fear memory in rats, offering a new strategy for PTSD [62].

CB1Rs exhibit surface (cell membrane) labeling, which often corresponds to synaptic contact sites [41], and broadly show intracellular localization [47]. Upon agonist stimulation, the receptors undergo endocytosis and are targeted for degradation [47]. CB1R activity and expression are regulated by desensitization or endocytosis [47, 63]. Similar modifications are due to the activation of mGluRs. Therefore, it is worth considering whether there are sites in the membrane (membrane mechanisms) and downstream mechanisms that allow CB1 and mGlu receptors to interact with each other, resulting in altered responses at the behavioral level.

Although the CB2 receptor is primarily localized in peripheral tissues, it may still play a role in the mechanisms described [51, 64]. CB2 receptors share only 44% amino acid sequence identity with CB1 receptors. However, their expression in macrophages, as well as in the soma and dendrites of neurons, microglia, and astrocytes, supports this hypothesis [51, 64]. THC has been documented to act as both an agonist and antagonist of CB2 receptors [51]. Given that CB2 receptors are mainly expressed in immune cells, their potential involvement in immune mechanisms related to depression or cognitive function cannot be ruled out [65, 66].

### Mechanisms of reciprocal regulation or interdependence of cannabinoid CB1 and metabotropic mGlu receptors. Are there interactions?

There is sparse literature on mutual regulation at the behavioral level between CB1Rs and mGluRs (PubMed search). Most available literature focuses on electrophysiological changes, synaptic plasticity, and Glu uptake [67–71]. However, the interaction at the level of CB1Rs and mGluR5 has been widely documented [67, 71]. The latest finding of Xiang et al. [71] using VU0092273 and VU-29 (a mGluR5 PAMs) demonstrated that the strengthening of LTP is mediated by GABA-ergic disinhibition mediated by eCB signaling. Suppression of inhibitory postsynaptic currents (eIPSCs) in the hippocampus (CA1) mediated by both PAMs was blocked by CB1Rs antagonist AM251 [71]. Furthermore, the above interaction was critical for fear conditioning in mice [71]. Similar mGluR5/CB1Rs interactions have been found for long-term depression (LTD) [67, 68]. It has been documented that transient depression of excitatory synaptic transmission is mediated by mGluR1/5-dependent eCB release, which inhibits transmission through presynaptic CB1Rs [67]. The above mechanism may be involved in anxiety and addiction [67]. A similar mechanism was previously described by Rouach and Nicoll [68]. The interaction between mGluR5 and CB1Rs was confirmed using a mGluR5 antagonist (MTEP) and a CB1Rs antagonist (rimonabant) in studies of memory and anxiety in animals [72]. In a series of experiments in rats, the authors found that MTEP effectively abolished anxiogenic effects mediated by rimonabant [72]. In contrast, rimonabant was unable to block the anxiety-like effects mediated by MTEP [72]. Moreover, rimonabant effectively inhibited memory impairment mediated by MTEP in a contextual conditioning paradigm [52]. The above interaction between mGluR5 and CB1Rs affects the release and uptake of Glu, as documented in slices of the striatum [70]. In addition, neuroprotective mechanisms involving mitogen-activated protein kinase (MEK)/extracellular-signal-regulated kinase (ERK1/2) and phosphatidylinositol-3 kinase (PI3K)/AKT proteins have been described for mGluR5/CB1Rs interactions [73].

Looking for interactions between CB1Rs and mGluRs of the other groups besides mGluRI, it is necessary to mention several studies by Barbara et al. [74]. The authors looked for interactions between CB1Rs and mGluRII in the rat prefrontal cortex using DCGIV (mGluRII agonist) and MSOPPE (mGluRII antagonist) [74]. The interaction between mGluRII and CB1Rs in a long-lasting depression of proximal synapses, in a postsynaptic activation of ERK, and synaptic plasticity were documented [74]. The authors did not find interactions in high-frequency stimulations (HFS)-dependent synaptic plasticity [74]. Considering mGluRIII, there is no data documenting the interaction between CB1Rs. We discovered that such interaction may exist in anxiety. Among others, when injecting AM251 and mGluR7 ligand, colocalization changes were detected in the mouse brain (manuscript in preparation).

One has to wonder what the mechanism of interaction between CB1R and mGluRs in the brain might be. The interactions will depend on the structure and functions performed. Both receptors are located presynaptically, i.e., quite close to each other for interaction. No “contact” sites between the two proteins have yet been discovered, but considering their colocalization, it points to two possibilities. First, the two receptors likely come into direct contact but may also interact via 5-HT1A receptors located presynaptically. The literature has shown the effects of both CB1R ligands on 5-HT1A [75] and mGluRs [76]. However, as of today, this is only a hypothesis and requires several studies to delve deeper into the topic.

Considering these all, there are interactions at the functional level and the communication of cellular signals, which do not infrequently possess contradictory effects when considering the reciprocal regulation between mGluRs and CB1. Functional interactions linking these two receptors take place at the level of the modulation of synaptic transmission, endocannabinoid signaling, synaptic plasticity, and neuronal excitability [77, 78]. Both mGluRs and CB1 receptors can modulate synaptic transmission by regulating the release of neurotransmitters. CB1 activation generally inhibits the release of excitatory neurotransmitters like Glu and inhibitory like GABA, while mGluRs can have varying effects depending on their type and location [77, 78]. Activation of group I mGluRs results in an increase in intracellular calcium levels, which can facilitate the release of neurotransmitters. This effect can be modulated by CB1R activity, as endocannabinoid signaling may counteract the excitatory actions of mGluRs by inhibiting Glu release [78].

Similarly, when considering signaling at interplay, mGluRs produce endocannabinoids [78]. Activating mGluRs in group I can stimulate the production of 2-AG, which acts retrogradely to activate presynaptic CB1Rs [79]. This activation inhibits the release of neurotransmitters, creating a feedback mechanism to modulate synaptic activity [79]. Conversely, groups II and III of mGluRs, which inhibit AC and reduce cAMP levels, can indirectly affect endocannabinoid signaling by modulating the enzymes involved in endocannabinoid synthesis and degradation [80]. Interactions at the synaptic plasticity level have been described in-depth in this review. LTP and LTD are processes underlying synaptic plasticity. Both receptors play a significant role in these processes. Group I mGluRs are involved in the induction of LTD through mechanisms that involve the production of endocannabinoids and subsequent activation of CB1Rs [81]. On the other hand, CB1R activation can modulate LTP by influencing the release of neurotransmitters and altering the synaptic strength. The balance between mGluRs and CB1Rs signaling is crucial for maintaining synaptic plasticity and proper neural circuit function [82]. All these indicated mechanisms do not exclude the possibility of hetero-dimer formation between mGluRs and CB1Rs, which can lead to the formation of receptor complexes that exhibit unique functional properties different from those of the individual receptors. This interaction can influence signaling pathways, receptor pharmacology, and physiological outcome.

## Conclusive remarks

There are few studies on the interaction between mGluR and CB1Rs on cognitive and mood changes. It is known and accepted that a decrease in presynaptic Glu release in the cortex is observed during CB1R activation. Similar observations have been made for the hippocampus. A large number of results indicate that CB1Rs are involved in depression, anxiety, and memory, but studies of mechanisms involving mGluRs are mainly limited to mGluR5. It is essential to look for reciprocal regulation between eCBs and other mGluRs of the mGluRII and mGluRIII groups to understand the background of these conditions and try to eliminate the side effects for which ligands of both receptor types are known. Both functional and signaling interactions between mGluRs and CB1Rs involve a sophisticated network of intracellular pathways that regulate neurotransmitter release, synaptic plasticity, and neuronal excitability. These interactions are mediated through the production of second messengers, modulation of ion channels, and retrograde signaling by endocannabinoids. Understanding these mechanisms provides insight into their roles in brain function and potential therapeutic targets for neurological and psychiatric disorders. However, further research using new methods is needed to fully elucidate the implications of CB1R and mGluR heteromers in health and disease.

## Abbreviations

AEA: Anandamide
AFC: Auditory fear conditioning
AD: Alzheimer’s disease
2-AG: 2-arachidonoylglycerol
BDNF: Brain-derived neurotrophic factor
CA1: Hippocampus CA1
CBD: Cannabidiol
CB1Rs: CB1 cannabinoid receptors
CPP: Conditioned place preference (morphine-induced)
DAG: Diacylglycerol
eCBs: Endogenous cannabinoids
eIPSCs: Inhibitory postsynaptic currents
EPM: Elevated plus maze
ERK: Extracellular receptor kinase
ERK1/2: Mitogen-activated protein kinase (MEK)/extracellular-signal-regulated kinase
FAAH: Fatty acid amide hydrolase
FST: Forced swim test
GABA: γ-aminobutyric acid
GIRK: G-protein-coupled receptor kinases
Glu: Glutamate
GPCRs: G protein-coupled receptors
iNOS: Inducible nitric oxide synthase
IP_3_: Inositol 1,4,5-triphosphate
LTD: Long-term depression
MAPKs: Mitogen-activated kinases
mGluRs: Metabotropic glutamate receptors
MTOR: Mammalian target of rapamycin
NAMs: Negative allosteric modulators
NMDARs: N-methyl-D-aspartate receptors
OBX: Olfactory bulbectomy
ORT: Object recognition test
PA: Passive avoidance
PAG: Rat dorsolateral periaqueductal gray
PAMs: Positive allosteric modulators
PI3K: Posphatidylinositol-3 kinase
PKC: Protein kinase C
PLA2: phospholipase A2
PLC: Phospholipase C
PLD: Phospholipase D
PO: Per os
SIH: Stress-induced hyperthermia
SPT: Sucrose preference test
TNF-α: Tumor necrosis factor-alpha
TST: Tail suspension test
UMS: Unpredictable mild stress
VFD: Venus flytrap domain.
